# Directing the oxidative folding of disulfide-rich peptides for enhanced engineering and applications

**DOI:** 10.1039/d5sc05617a

**Published:** 2025-09-30

**Authors:** Xueting Cheng, Chuanliu Wu

**Affiliations:** a Department of Chemistry, College of Chemistry and Chemical Engineering, The MOE Key Laboratory of Spectrochemical Analysis and Instrumentation, Xiamen University Xiamen 361005 P. R. China chlwu@xmu.edu.cn

## Abstract

Disulfide-rich peptides (DRPs) leverage dense disulfide networks to form rigid and stable cores, enabling exceptional proteolytic resistance and precise target complementarity. These attributes drive their utility as high-affinity molecular tools in bioanalytics/chemical biology and clinically validated therapeutics (*e.g.*, ziconotide for chronic pain and insulin for diabetes). However, DRP functionality critically depends on native oxidative folding, where inefficient disulfide pairing causes low production yields, induces functional instability through disulfide isomerizations, and triggers misfolding upon sequence engineering. Recent advances in directed oxidative folding permit precise pathway control, facilitating efficient engineering and discovery of functional DRPs, thereby accelerating diagnostic and therapeutic development. Herein, we summarize novel strategies that actively direct the oxidative folding of DRPs to enhance their engineering and applications. Additionally, we present our perspective on key challenges in DRP design and discovery associated with oxidative folding, and propose future research directions to advance this field.

## Abstract 2

Oxidative folding of DRPs poses a major challenge to their design, engineering, and application. This perspective introduces methods to actively direct peptide oxidative folding, which enhance their utility and enable the discovery of novel DRPs.

## Graphical abstract

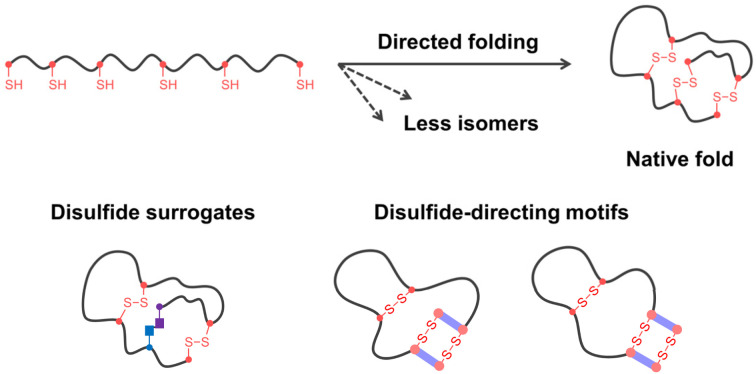

## Introduction

Disulfide-rich peptides (DRPs) constitute a remarkable class of biomolecules, distinguished by their dense network of disulfide bonds.^1–9^ This defining structural feature forms a covalently cross-linked, rigid core that locks the peptide into a highly stable and precise three-dimensional fold. The exceptional conformational stability of DRPs underpins their unique value by conferring strong resistance to enzymatic degradation in physiological environments and enabling precise structural complementarity with target protein surfaces.^10–12^ Consequently, DRPs exhibit outstanding binding specificity and affinity, propelling their widespread adoption as powerful recognition elements in bioanalytics, chemical biology, and biomedical research.^13,14^ Critically, this inherent stability and targetability also establish DRPs as a clinically validated therapeutic modality, with notable examples like ziconotide, linaclotide, and insulin already benefiting patients.^15–17^ Thus, these attributes make DRPs uniquely versatile, serving effectively both as molecular tools and as promising therapeutic candidates and drugs.

However, realizing the full potential of DRPs critically depends on the correct pairing of their disulfide bonds—a process known as oxidative folding.^18–21^ The efficiency and fidelity of this folding process are critical. Low folding efficiency leads to synthetic challenges, high production costs, and reduced yields. Moreover, inefficient folding increases the risk of disulfide bond scrambling.^19,22–24^ Natively-folded DRPs may undergo disulfide isomerizations in complex biological environments, compromising their functional stability.^22,25^ This challenge becomes particularly acute when DRPs are engineered as molecular scaffolds to confer new functions. Modifications to the peptide sequence—essential for developing novel binders or therapeutics—frequently perturb the delicate energy landscape of oxidative folding, altering folding pathways and often leading to misfolded, inactive products.^23,24,26^ Controlling these oxidative folding pathways thus represents a fundamental hurdle in the rational engineering and development of new DRPs.

Recent innovations in directing oxidative folding have advanced the design, engineering, and discovery of DRPs through two major approaches: chemical engineering of disulfide surrogates and strategic encoding of disulfide-directing motifs.^2,6,27^ For instance, diselenide bonding leverages selenocysteine's rapid bond formation and thermodynamic dominance to override sequence-encoded folding cues.^28^ Non-reducible mimetics (*e.g.*, thioethers) enforce oxidative folding pathways through geometrically isosteric, chemically inert crosslinks that eliminate disulfide scrambling.^29^ Complementing these strategies, disulfide-directing motifs exploit inter-cysteine spacers to preorganize disulfide pairing, enabling precise disulfide connectivity independent of global sequence context.^2^ Their intrinsic disulfide-pairing properties decouple oxidative folding from primary sequences, permitting combinatorial DRP library construction and screening in vast sequence space.^2^ These approaches provide rational control over oxidative folding pathways, enabling oxidative folding of DRPs with enhanced stability and minimal isomerization. In this Perspective, we first discuss the inherent complexities of peptide oxidative folding and elucidate why precise folding pathway control remains a central challenge for DRPs. We then highlight key methods for actively directing the oxidative folding of DRPs through case studies of chemical surrogates, disulfide-directing motifs, and some other emerging strategies. Additionally, we discuss combinatorial folding strategies that enable precise control over DRP folding and expand the structural diversity accessible for functional DRP discovery. Finally, we discuss key opportunities and challenges in expanding the function and application of DRPs.

## Oxidative folding of DRPs

**Fig. 1:**
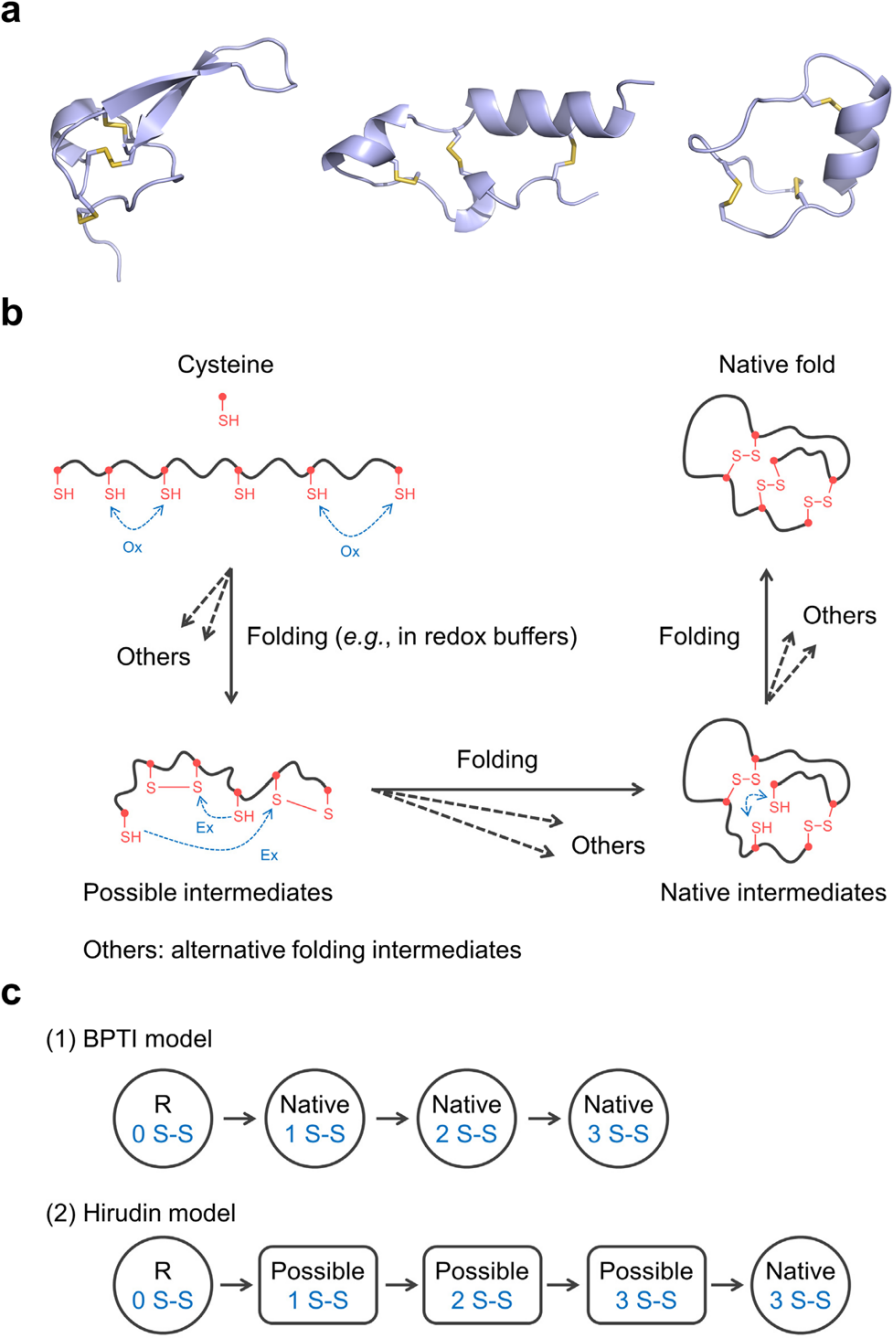
(a) Representative peptide structures (PDB IDs: 1AXH, 9J5H, 7W8K, respectively, from left to right; disulfide bonds are shown in yellow) stabilized by multiple disulfide bonds. (b) Simplified pathways of oxidative folding for DRPs. The oxidative folding of peptides and proteins *in vitro* is often performed in redox buffers containing oxidized glutathione (GSSG). Ox: oxidation; Ex: exchange. (c) Two extreme models of folding pathway for proteins with three disulfide bonds. R: reducing polypeptides; Native: native intermediates or final products; Possible: possible intermediates; S–S: disulfide bonds.

In biological systems, proteins and peptides containing disulfide bonds are synthesized as linear polypeptide chains on ribosomes and subsequently fold into their functional three-dimensional structures through oxidative folding.^30^ This process involves the oxidation of cysteine thiol groups to form disulfide bonds, which play a crucial role in stabilizing the native structures.^18,31^ Disulfide bonds are particularly abundant in secretory proteins, and smaller proteins densely packed with multiple disulfides are collectively termed as DRPs. The accurate formation of specific disulfide pairings is essential for both the structural integrity and biological activity of these molecules (Fig. 1a).^32,33^ Improper disulfide connectivity can lead to polypeptide misfolding, aggregation, and eventual degradation by the cell's proteolytic systems. To prevent such outcomes, oxidative folding in the cell is tightly regulated by a complex folding machinery composed of molecular chaperones, oxidoreductases such as protein disulfide isomerase (PDI), and redox buffering agents, primarily the glutathione redox couple (GSH/GSSG).^34,35^ Only natively folded molecules pass the quality control system and are allowed to proceed to secretion, whereas misfolded species are efficiently recognized and targeted for degradation.^36–38^

While cells have evolved efficient machinery to fold even complex DRPs, reproducing this fidelity in synthetic environments remains a significant challenge. The short length of DRPs reduces sequence-based stabilizing interactions and folding propensity, making it more difficult to guide the peptide chain toward its native structure (Fig. 1b).^2^ Moreover, the combinatorial complexity of disulfide bonding further complicates the folding landscape.^39^ For example, a peptide with six cysteine residues (forming three disulfide bonds) can form 15 distinct isomers, while a peptide with eight cysteines (forming four disulfide bonds) can theoretically form 105. This combinatorial complexity is further amplified by the existence of kinetically trapped folding intermediates bearing non-native disulfide bonds, increasing the heterogeneity of species formed during the folding process.^19,40^ Oxidative folding proceeds through a cascade of thiol-disulfide exchange reactions, which are bimolecular nucleophilic substitution processes (Fig. 1b).^39^ These reactions initiate when a thiolate anion attacks the sulfur atom of an existing disulfide bond. This nucleophilic attack cleaves the disulfide bond, generating a new thiolate anion that propagates the reaction cascade. This cycle continues iteratively until the thermodynamically most stable native structure, defined by a unique set of native disulfide bonds, is achieved. However, due to the stochastic nature of these processes, folding in cell-free systems often results in low yields of the desired native structure.

Although the oxidative folding pathways of short DRPs have been less systematically explored, extensive studies on disulfide-rich proteins have revealed that their folding processes exhibit a high degree of complexity and diversity, often involving heterogeneous ensembles of intermediates.^39,41–43^ These pathways can be broadly classified into two major types according to the nature of the intermediates (Fig. 1c).^44^ In one type, folding proceeds through a limited set of intermediates composed exclusively of native disulfide bonds. In the other, folding involves a large and heterogeneous collection of isomers with varying numbers and arrangements of disulfide bonds. Bovine pancreatic trypsin inhibitor (BPTI) and hirudin represent two prototypical proteins that exemplify these contrasting folding strategies,^45,46^ providing important paradigms for understanding oxidative folding in disulfide-rich proteins. Many other disulfide-rich proteins also adopt a hybrid BPTI–hirudin folding model, in which native and non-native intermediates form concurrently during folding (*e.g.*, TAP: tick anticoagulant peptide).^41,47^ Notably, insights gained from these well-characterized protein folds highlight the delicate balance between pathway restriction and conformational heterogeneity, offering valuable perspectives for elucidating the folding behavior of smaller DRPs.

The broad distribution of naturally occurring DRPs, spanning venomous animals (*e.g.*, conotoxins), plants, and humans (*e.g.*, defensins), reflects their extensive sequence and structural diversity.^3,14,48^ This diversity has spurred persistent research into optimizing *in vitro* oxidative folding for DRP synthesis and engineering. Common optimization strategies involve careful manipulation of environmental parameters such as pH, temperature, ionic strength, and the composition of redox buffers—typically using specific GSH/GSSG ratios.^19,20,49^ In addition, the use of organic solvents or denaturants can enhance peptide solubility, stabilize folding intermediates, and ultimately promote native folding.^50,51^ While empirical optimization enables efficient folding for some DRPs, many sequences prove refractory to correct folding *via* direct oxidation. These peptides typically yield low quantities of the native product while generating intractable mixtures of disulfide-scrambled isomers. To ensure precise disulfide connectivity, orthogonal chemical strategies have been developed. These involve the selective protection of cysteine residues with orthogonally removable protecting groups during peptide synthesis,^52–56^ including the recent introduction photocleavable protecting groups.^57,58^ Specific cysteine pairs are then sequentially deprotected and oxidized in a controlled, stepwise manner. This approach bypasses the stochastic nature of direct oxidative folding, ensuring the formation of predetermined disulfide bonds. However, these orthogonal methods are complex, time-consuming, and substantially increase synthesis costs. More critically, they are incompatible with ribosomal synthesis and biological display technologies such as phage and yeast display, precluding their use in high-throughput DRP library generation and screening.

Achieving efficient and reliable oxidative folding remains a central goal in chemical research of DRPs. High folding efficiency is vital not only for the cost-effective production and functional optimization of existing DRPs, but also for enabling the rational design and discovery of novel sequences with customized biological activities. Without robust folding pathway control, the exploration of the vast sequence and structural diversity required for next-generation DRP development is severely limited. Therefore, understanding the complex mechanisms underlying oxidative folding and developing methods to guide this process continue to pose key challenges and constitute an active area of research. In the following sections, we highlight recent advances in strategies aimed at actively directing the oxidative folding of DRPs.

## Strategies to direct oxidative folding

### Chemical engineering of disulfide surrogates

**Fig. 2:**
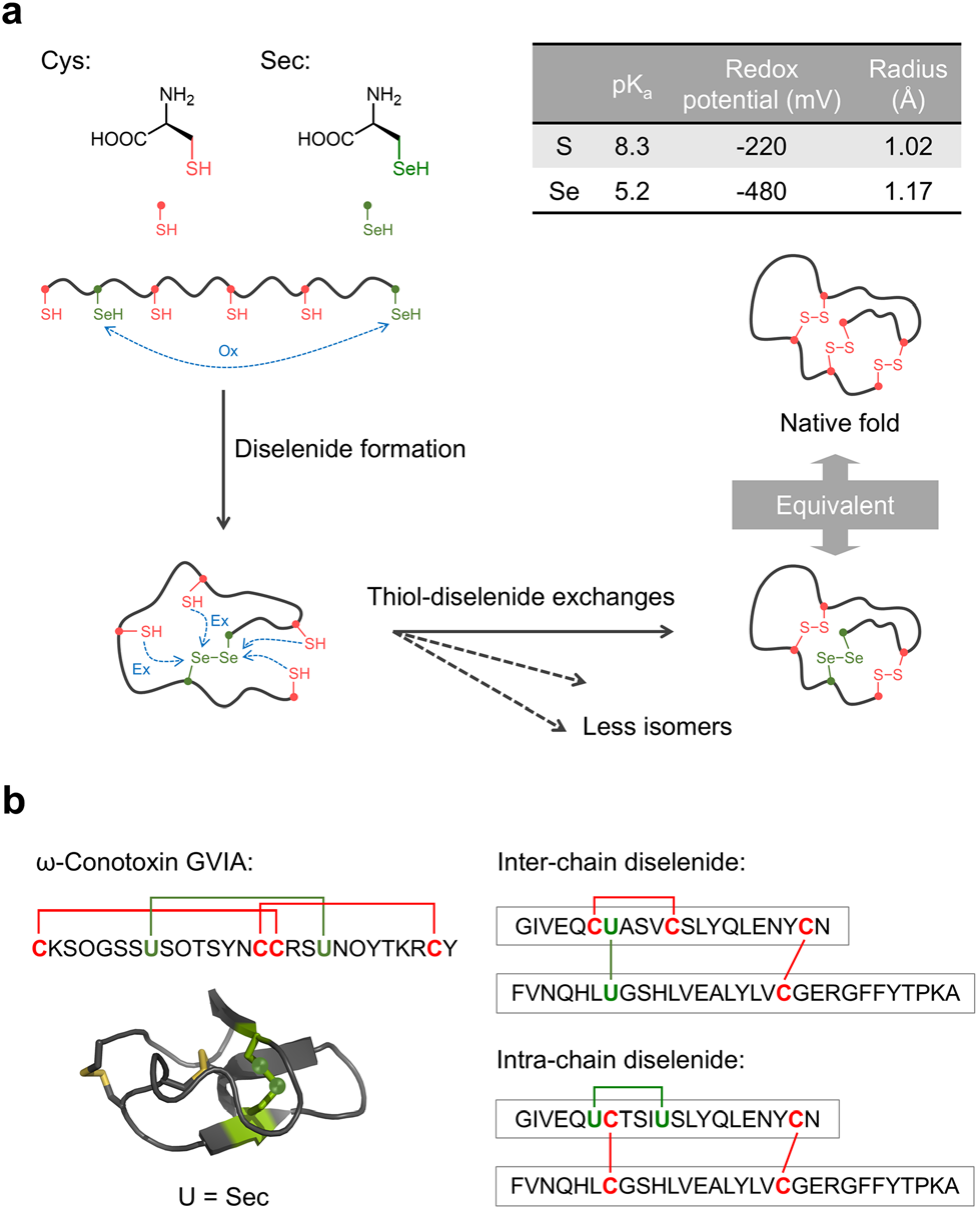
(a) Structures and physical parameters of Cys and Sec, and selenocysteine-directed oxidative folding of DRPs. Ox: oxidation; Ex: exchange. (b) GVIA conotoxin with the incorporation of a diselenide bridge surrogate and two selenol-insulins with inter-chain or intra-chain diselenide bridges.

Selenocysteine (Sec) shares structural similarities with cysteine but also exhibits distinct chemical properties.^28,59–63^ Like cysteine, Sec can undergo oxidation to form covalent linkages—specifically, diselenide bonds analogous to disulfide bonds (Fig. 2a). Due to the similar atomic radius of sulfur and selenium, diselenide and disulfide bonds possess comparable lengths. However, a key distinction lies in the significantly lower p*K*_a_ of the selenol group in Sec compared to the thiol group in cysteine. This results in a higher proportion of the reactive selenolate species at physiological pH. Furthermore, diselenide bonds exhibit a substantially lower redox potential than disulfide bonds, promoting both more rapid bond formation and increased thermodynamic stability. These properties make Sec an effective tool for modulating oxidative folding pathways in DRPs and proteins.^61,64–70^ Early studies demonstrated that replacing disulfides with diselenide in peptides can yield selenol-analogues that preserve native structure and function while enhancing stability.^59,62,71–74^ Moreover, strategic substitution of cysteine pairs with selenocysteine residues has been shown to direct folding toward native disulfide connectivity while minimizing structural perturbations (Fig. 2a).^74–77^

This strategy has been effectively demonstrated in selenocysteine-substituted analogues of naturally occurring conotoxins, such as ω-conotoxin GVIA and μ-conotoxin SIIIA (Fig. 2b).^28^ In these cases, diselenide incorporation enables oxidative folding to proceed without exogenous oxidants. The mechanism underlying this autocatalytic folding involves three key steps: (i) the diselenide bond transfers an oxidizing equivalent to other cysteine residues, forming a disulfide bond; (ii) the resulting selenolate promotes disulfide isomerization *via* nucleophilic attack; and (iii) molecular oxygen reoxidizes the selenols, regenerating the diselenide. This redox cycling accelerates the folding and supports efficient native disulfide formation. This diselenide substitution approach has also been applied to more complex and clinically important DRPs such as insulin, a two-chain hormone stabilized by three intra- and inter-chain disulfide bonds (Fig. 2b).^78–80^ For instance, replacing the inter-chain CysA7–CysB7 disulfide bond with a diselenide has been shown to enhance folding efficiency and enzymatic stability, while preserving native conformation and biological activity.^78,79^ Similarly, substitution of the intra-chain CysA6–CysA11 disulfide bond with a diselenide facilitates folding by accelerating inter-chain association and enhancing resistance to both reductive and proteolytic degradation.^80^ Notably, insulin folding presents a particularly challenging case due to the requirement for precise inter-chain alignment and the selective formation of inter-chain disulfide bonds, which are often kinetically disfavored. The ability of diselenide substitution to overcome these inherent barriers highlights its robustness and broad applicability in directing oxidative folding across diverse DRP scaffolds.

**Fig. 3:**
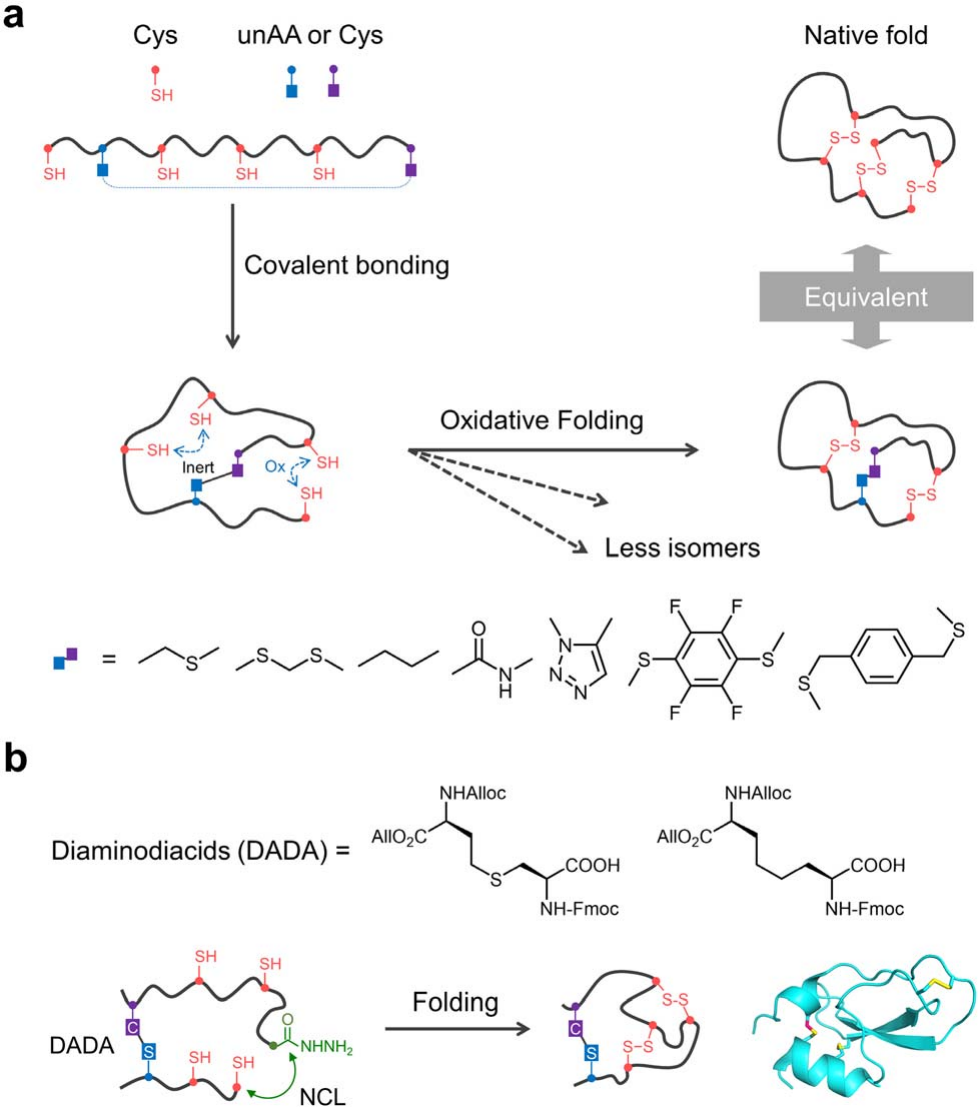
(a) Oxidative folding of DRPs with non-reducible disulfide mimetics. unAA: unnatural amino acid; Ox: oxidation. (b) DRPs with cystathionine surrogates synthesized by NCL-assisted DADA approach and their structures (PDB ID: 6KZF).

In addition to the use of dynamic diselenide bonds, the strategic incorporation of nonreducible disulfide surrogates has also emerged as a powerful strategy for modulating the oxidative folding of DRPs (Fig. 3).^27^ These chemically more stable crosslinks offer enhanced resistance to reductive cleavage and, importantly, suppress disulfide scrambling by reducing the lability of remaining native disulfide bonds. Their utility lies in their capacity to mimic the isosteric geometry of native disulfide bonds, although the fidelity of this mimicry varies depending on the specific surrogate employed. Remarkably, the replacement of even a single disulfide bridge with a nonreducible analogue can dramatically simplify the folding pathways.^29^ For example, substituting one disulfide bond in a peptide with three disulfide bonds reduces the number of potential disulfide isomers from fifteen to just three. This predefined connectivity can effectively eliminate misfolded regioisomers and promote efficient folding into the native, bioactive conformation.

The effectiveness of nonreducible mimetics stems from their capacity to impose structural constraints comparable to native disulfide bonds while resisting redox-mediated cleavage (Fig. 3a).^27^ Among sulfur-containing surrogates, several notable variants have been developed, each with distinct structural implications. Thioether bridges that are characterized by the absence of one sulfur atom relative to disulfides (a structural feature observed in lanthionines) provide substantial stability under reducing conditions but feature a shortened bridge length.^81^ Conversely, thioacetals constitute another class whose application benefits from efficient methyl iodide-based alkylation methods,^82^ yet they incorporate an additional methylene group that extends bridge length.^83,84^ Thus, both lanthionine and thioacetal bridges exhibit non-native bond lengths relative to natural disulfides which might cause local conformational perturbations in tightly folded peptide regions.^85^ Importantly, however, deviations in bond length do not invariably compromise overall structure or activity: for instance, incorporation of a methylenethioacetal into BPTI conferred enhanced stability while preserving binding affinity and biological function.^84^ By comparison, cystathionine analogues provide an even closer mimic, with the replacement of one sulfur by a methylene group yielding near-ideal replication of native disulfide geometry and bond length.^86^ This precise isostericity makes cystathionine surrogates particularly promising for minimizing structural perturbations while maintaining stability. In contrast to thioether and thioacetal surrogates, dicarba linkages provide an alternative that preserves disulfide-like geometry without introducing heteroatoms.^87^ Despite being entirely sulfur-free, dicarba bridges can be engineered to closely mimic the spatial arrangement of native disulfides, acting as structurally faithful and chemically inert crosslinkers. This combination of geometric precision and strong resistance to chemical degradation makes dicarba surrogates especially advantageous for biological applications where both conformational integrity and long-term stability are critical. Other covalent linkages, including triazole bridges, amide bonds, perfluoroaromatic substitutions, and xylene linkages, have also been explored as stable replacements for disulfide bonds.^88–94^ However, these linkages usually do not preserve the native conformation of peptides, and the resulting bioactivity is largely determined by the extents of structural perturbation that the peptide can tolerate.

The application of these mimetics has been greatly advanced by innovations in synthetic methods, particularly the development of diaminodiacid (DADA) building blocks.^29,95^ Initial strategies relied on solid-phase peptide synthesis (SPPS) using preassembled DADA units, which proved only effective for installing mimetic bridges within short loops. These approaches faced limitations in constructing longer-range or solvent-exposed bridges. Interestingly, this limitation can be addressed by integrating native chemical ligation with DADA chemistry (Fig. 3b).^96^ The NCL-assisted DADA approach facilitates intramolecular cyclization in solution, circumventing the steric constraints of on-resin strategies. This has enabled the construction of peptides with surrogate bridges spanning up to 50 residues, as demonstrated in the synthesis of mimetics of μ-conotoxin KIIIA, spider toxin Hm-3, and snake toxin CaC, yielding analogues that closely recapitulate the native structure. Therefore, by combining precise structural mimicry—as seen in cystathionine and dicarba analogues—with enhanced chemical robustness, and employing novel synthetic approaches such as DADA chemistry and NCL-assisted cyclization, researchers can engineer peptides with improved folding efficiency, stability, and bioactivity. These advances not only deepen our understanding of peptide folding mechanisms but also expand the capabilities of DRPs for functional applications. As synthetic methodologies progress, strategic use of nonreducible disulfide mimetics will remain critical for developing DRP-based molecular tools and therapeutics.

**Fig. 4:**
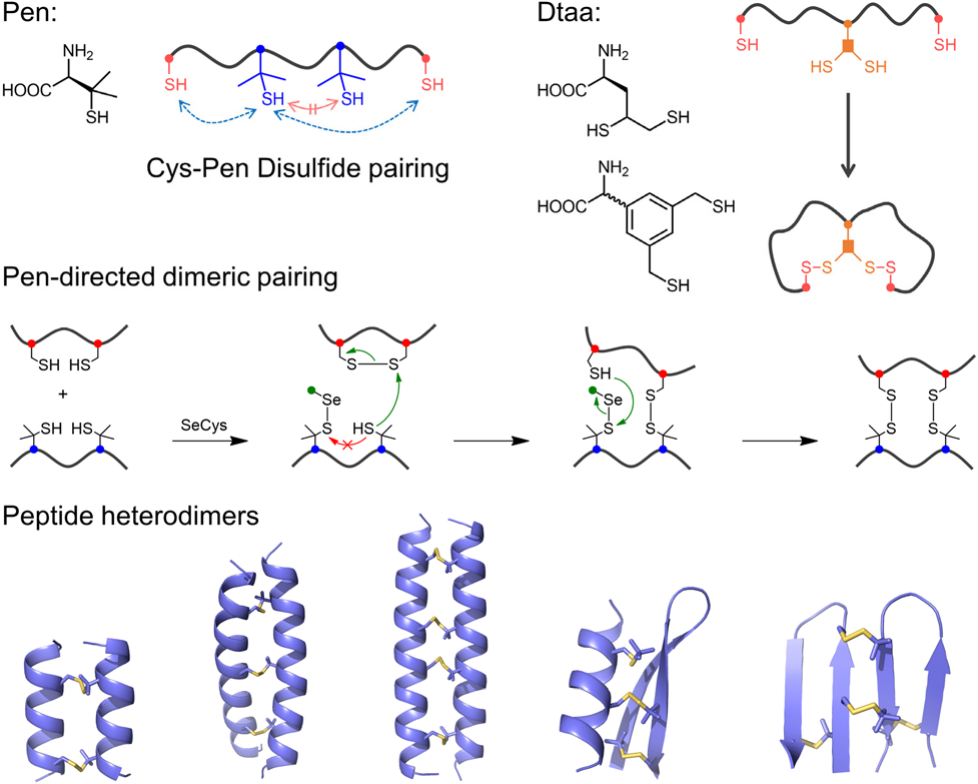
Pen or dithiol-bearing amino acid-directed disulfide pairing and folding of peptides, and computationally designed peptide heterodimers with multiple Cys-Pen disulfide bonds (PDB IDs for the two designs on the right: 7FB8 and 7FBA).

Beyond conventional disulfide surrogates, thiol-containing noncanonical amino acids—such as dithiol-containing residues and the β,β-dimethyl-substituted cysteine analogue penicillamine (Pen)—have also been developed as promising tools for guiding the oxidative folding pathways of DRPs.^22,25,97–99^ These residues can form non-native disulfide bonds that structurally mimic natural cystine linkages. By selectively forming heterodisulfide bonds with cysteine residues, they exert a strong influence on disulfide pairing and, consequently, on folding pathways. Penicillamine exemplifies this strategy (Fig. 3c). Due to the steric hindrance introduced by its two methyl groups at the β-carbon, the formation of stable Pen–Pen disulfide bonds is kinetically disfavored. Instead, Pen exhibits a strong preference to form heterodisulfide bonds with cysteine and other less-sterically hindered thiols during oxidation.^25,97,98^ When incorporated into peptide sequences alongside an equal number of other thiols, this selective pairing drives the oxidative folding process toward defined disulfide connectivities, enabling the construction of specific bicyclic or tricyclic architectures.^25,98,100^ Building on the high specificity of the Pen-mediated disulfide pairing, we recently developed a general strategy for the design and synthesis of disulfide-bridged peptide heterodimers (Fig. 4).^99^ This approach combines directed disulfide pairing chemistry with computational *de novo* design. The chemical component exploits the efficient formation of multiple interchain Cys–Pen disulfide bonds, facilitated by selenol-l-cystine (SeCys) as a redox mediator. Computational modeling ensures the structurally compatible integration of this pairing chemistry into designed peptide scaffolds, yielding dimeric assemblies with orthogonal association properties and high folding efficiency (Fig. 4). Similarly, dithiol-containing amino acids can be strategically employed to control disulfide pairing within peptides.^22,25^ Due to conformational or spatial constraints, the two thiol groups in a single dithiol residue are generally unable to form an internal disulfide bond. As a result, they preferentially form disulfide bridges with other thiol-containing residues in the peptide chain, allowing precise regulation of disulfide connectivity and folding pathways (Fig. 4). Altogether, the incorporation of these noncanonical thiol-bearing amino acids facilitates the engineering of DRPs with enhanced oxidative folding efficiency and reduced susceptibility to thiol-mediated disulfide reshuffling. This strategy offers a promising platform for controlling peptide folding pathways. Notably, the high specificity of Cys–Pen disulfide pairing can be further combined with complementary fold-regulating strategies to direct the oxidative folding of peptides with random sequences to design various multicyclic peptides, as detailed later.

### Strategic encoding of disulfide-directing motifs

Chemical engineering strategies employing disulfide surrogates to direct oxidative folding exhibit inherent limitations that constrain their broader applicability. Primarily, though they have been successfully applied to naturally occurring DRPs or close analogs with pre-defined structures, their effectiveness typically diminishes when confronted with highly diverse random sequences, a fundamental requirement in modern peptide discovery platforms reliant on combinatorial libraries.^101–104^ Furthermore, these strategies fundamentally depend on post-synthetic modifications or incorporation of unnatural amino acids (unAAs) to replace native cystine disulfide bonds.^27^ While genetic code reprogramming or expansion techniques can be used to introduce certain unAAs,^105–109^ the insertion of paired unAAs with functionable groups that can form disulfide surrogates under biocompatible conditions remains a formidable challenge. Moreover, no method currently allows for the direct incorporation of diaminodiacid residues into ribosomally expressed peptides, despite the fact that such mimetics offer intrinsic advantages in replicating the geometry of native disulfide bonds. Consequently, while powerful for optimizing specific known peptides, current surrogate-based methods are poorly suited for high-throughput library generation and screening technologies that require efficient and correct folding across vast arrays of diverse random sequences. This critical gap underscores the pressing need for alternative strategies capable of actively directing native cysteine pairing and oxidative folding without recourse to synthetic disulfide surrogates or unAA incorporation.

**Fig. 5:**
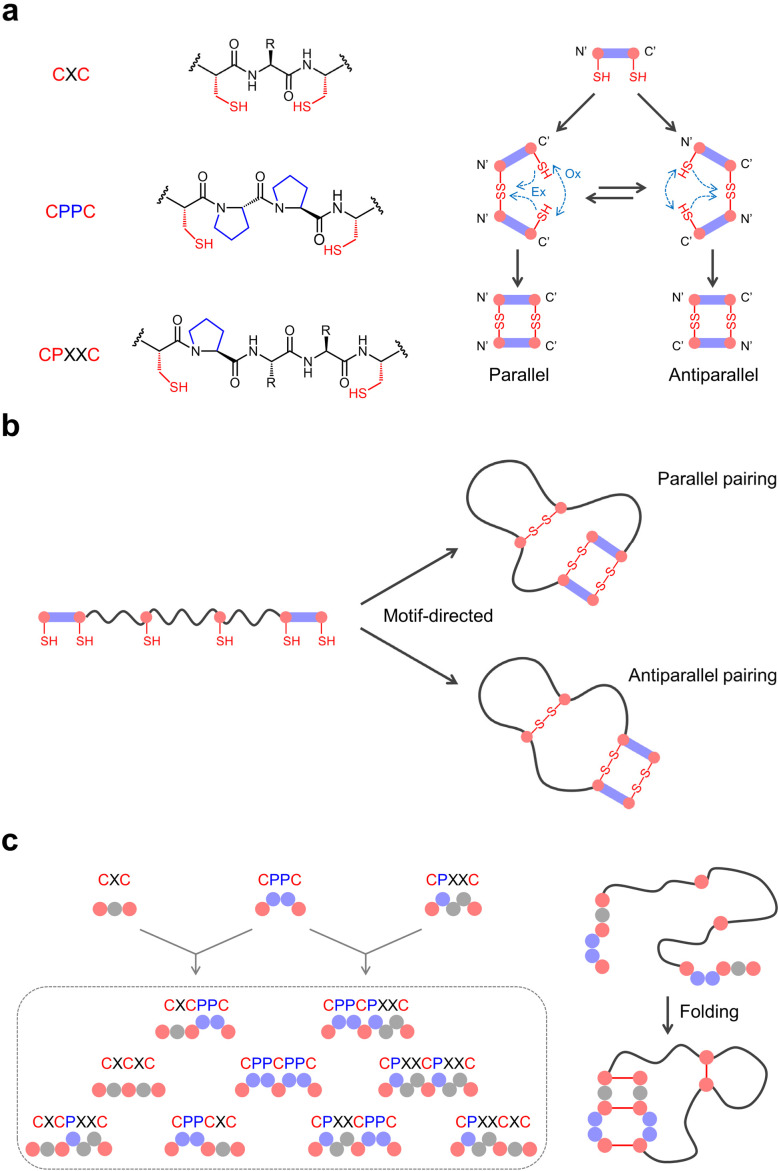
(a) Three biscysteine motifs and their disulfide pairing propensity to form parallel and antiparallel dimers. Ox: oxidation; Ex: exchange. (b) Oxidative folding of peptides with a pair of biscysteine motifs incorporated to form two different isomers. (c) Design of triscysteine motifs by tandem combination of biscysteine motifs, and the directed oxidative folding of a peptide with two triscysteine motifs.

To overcome these limitations, we have developed a generalized motif-directed oxidative folding strategy that encodes disulfide pairing information into short cysteine-containing peptide motifs, shifting the control of oxidative folding from global sequence to local structural elements.^2^ These motifs—such as CXC, CPPC, and CPXXC—possess intrinsic disulfide-directing tendencies, allowing peptides to fold spontaneously into multicyclic architectures under mild redox conditions (Fig. 5a).^6,98,110,111^ Particularly, this strategy eliminates the need for synthetic modifications or unAA incorporation and is fully compatible with ribosomal expression and display technologies. Our foundational work began with the minimal CXC motif, which we reported in 2012.^6^ Due to ring strain within the disulfide-closed macrocycle, CXC favors forming mixed disulfide bonds or dimeric structures. For example, when a peptide contains two CXC motifs, the cysteines from different motifs preferentially pair with each other to form inter-motif disulfide bonds, rather than producing intra-motif closed CXC disulfides. This feature enabled its use in peptides with four or six cysteines to direct disulfide pairing, albeit with modest yields. A major advancement came with the discovery of CPPC motif, where two cysteines are spaced by a proline–proline dipeptide.^110,112^ Similar to CXC, CPPC disfavors intra-motif disulfide formation, owing to the rigidity and extended conformation imposed by the proline residues. However, in contrast to CXC, peptides containing CPPC motifs show a pronounced preference for parallel dimeric pairing over antiparallel pairing. Moreover, when two CPPC motifs are incorporated into a single peptide, variation in the loop length between them revealed a striking effect on pairing geometry: longer loops favor parallel configurations, whereas shorter loops bias folding toward antiparallel arrangements due to conformational constraints imposed by the loop.

We later identified a more flexible class of motifs, CPXXC, which tends to form antiparallel disulfide pairing.^111^ Unlike CPPC, the CPXXC motif allows more sequence variability, with only the proline residue being conserved. This adaptability facilitates its integration into a wide variety of peptide structures. Peptides containing two CPXXC motifs typically oxidize to yield well-defined antiparallel products. These disulfide-directing motifs form the basis for constructing multicyclic peptide scaffolds with predictable folding outcomes. By strategically incorporating pairs of biscysteine motifs and free cysteines into random sequences, we can design disulfide-directed multicyclic peptides (DDMPs) containing up to three disulfide bonds (Fig. 5b) with exceptionally high oxidative folding efficiencies.^110,111^ The arrangement and spacing of cysteines control the final multicyclic topology depending on motif type and inter-motif loop length. For instance, peptides containing two CPPC motifs and two free cysteines separated by five-residue segments can form various tricyclic structures.^110^ Depending on the relative spacing, oxidation yielded high folding efficiency and either parallel or antiparallel CPPC pairing. Similar outcomes were achieved with CPXXC motifs, although they often favored antiparallel pairing exclusively.^111^

To further expand structural diversity and complexity, we have recently developed tandem triscysteine motifs by combining known biscysteine motifs into single motifs containing three cysteines (Fig. 5c).^113^ We examined their disulfide-pairing properties and found that, although in principle the three cysteines within each motif could adopt multiple alternative pairing patterns, these motifs consistently favored the formation of well-defined dimeric products, in which the motifs align in an orderly arrangement to form three inter-motif disulfide bonds. This intrinsic bias toward specific disulfide connectivity greatly simplifies oxidative folding outcomes. For instance, model peptides composed of primarily glycine, lysine, and tryptophan residues, incorporating two CPPCXC motifs together with two additional cysteines, folded predominantly into a single product with >90% yield. In these products, the two CPPCXC motifs paired in a parallel manner to generate three disulfide bonds, while the extra cysteine residues formed an additional disulfide linkage. Altogether, these findings show that the strategic encoding of biscysteine or triscysteine motifs into peptides allows for rational design of highly constrained, multicyclic peptide scaffolds with well-defined folding directions.

**Fig. 6:**
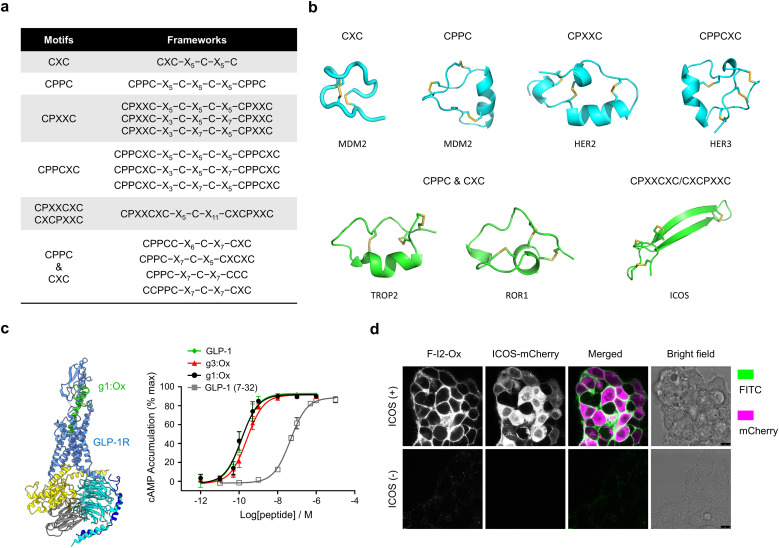
(a) DDMP libraries reported by our group. (b) Representative peptides selected from our peptide libraries with structures characterized by NMR. (c) Cryo-EM structure of g1:Ox-bound GLP-1R in complex with G_s_ and functional cAMP assay demonstrating the activity of g1:Ox. (d) Confocal fluorescence images of ICOS-expressing cells with ICOS-binding DDMP labeled with fluorophore.

Using DDMP as templates, we have constructed diverse peptide libraries using phage and yeast display technologies.^110,111,114–116^Fig. 6a and b summarized some peptide libraries that have been reported from our group for target screenings and representative multicyclic peptides characterized structurally using NMR. The first-generation library contained three disulfide bonds were constructed by the incorporation of two CPPC motifs (Fig. 6a). This library was screened first against MDM2, a key E3 ubiquitin ligase. Screening yielded a high-affinity binder (drp1, *K*_i_ = 23.7 nM) that folded into a well-defined tricyclic structure with two parallelly paired CPPC motifs.^110^ Structural analysis revealed a well-folded α-helix stabilized by a dimeric CPPC mini-loop (Fig. 6b).^112^ We next developed a CPXXC-based DDMP library by varying the spacing between cysteines to enhance topological diversity (Fig. 6a). This library has been screened against four cell-surface proteins: EphA2, FGFR1, HER2, and HER3.^111^ Selected binders exhibited nanomolar to micromolar affinities, and structural analysis confirmed the presence of well-folded products. Notably, peptides targeting FGFR1 and HER2 shared the same disulfide connectivity but differed in conformation, underscoring the structural plasticity of CPXXC motifs. More recently, we have applied motif-directed oxidative folding for the stabilization of long α-helical hormones such as GLP-1 (Fig. 6c).^117^ By embedding disulfide-directing motifs into the peptide backbone, we successfully stabilized α-helices with free N-terminus, which is essential for receptor binding and activation. The resulting DDMP agonist (g1:Ox) exhibited enhanced helicity, proteolytic resistance, and binding affinity compared to the native GLP-1 peptide. This study thus opens new avenues for engineering bioactive peptides for receptor modulation.

We have also developed libraries using triscysteine motifs.^113^ Library screening has identified binders with high affinity and specificity toward HER3 and ICOS. A HER3-binding peptide constrained by two CPPCXC motifs exhibited 56 nM affinity and adopted an α-helical structure (Fig. 6b). Another DDMP targeting ICOS displayed a β-sheet fold stabilized by antiparallel pairing between CPXXCXC and CXCPXXC motifs (Fig. 6b). These binders demonstrated high target selectivity on cell surfaces and could be used for the detection of ICOS expression on the cell surface (Fig. 6d). Additionally, using *de novo* cysteine frameworks discovered from our lab, we developed libraries with not defined disulfide connectivity to discover binders for TROP2 and ROR1 (Fig. 6b).^118^ One ROR1-targeting peptide, when labeled with fluorescein, enabled irreversible and high-sensitivity imaging of ROR1 at picomolar concentrations, facilitated by bivalent binding. To enhance affinity beyond what phage libraries allow, we transitioned to yeast display and developed a DDMP-evolving system. Starting from a CPPC-containing DDMP with submicromolar affinity for CD28, we used error-prone PCR and selection to evolve variants with picomolar binding affinity.^115^ These high-affinity binders were then engineered into fluorescent probes for visualization and analysis of CD28 on human T cells.

In summary, our development of disulfide-directing motifs has introduced a versatile, generalizable, and evolution-compatible strategy for directing the oxidative folding of DRPs. By leveraging local structural elements such as CPPC, CPXXC, and triscysteine motifs, we have overcome the inherent folding unpredictability and complexity that has long hindered the design and discovery of DRPs. This approach enables the construction of highly diverse, well-folded multicyclic peptides from randomized sequences without reliance on natural scaffolds or complex chemical synthesis. As a result, motif-directed oxidative folding opens new avenues for rational peptide design, high-throughput screening, and the development of peptide-based therapeutics, diagnostics, and molecular probes. As the field of synthetic and therapeutic peptides continues to evolve, motif-directed folding offers a foundational platform for creating next-generation DRPs that bridge the functional gap between small molecules and proteins, while also enables access to previously unexplored structures and functions.

### Other strategies

**Fig. 7:**
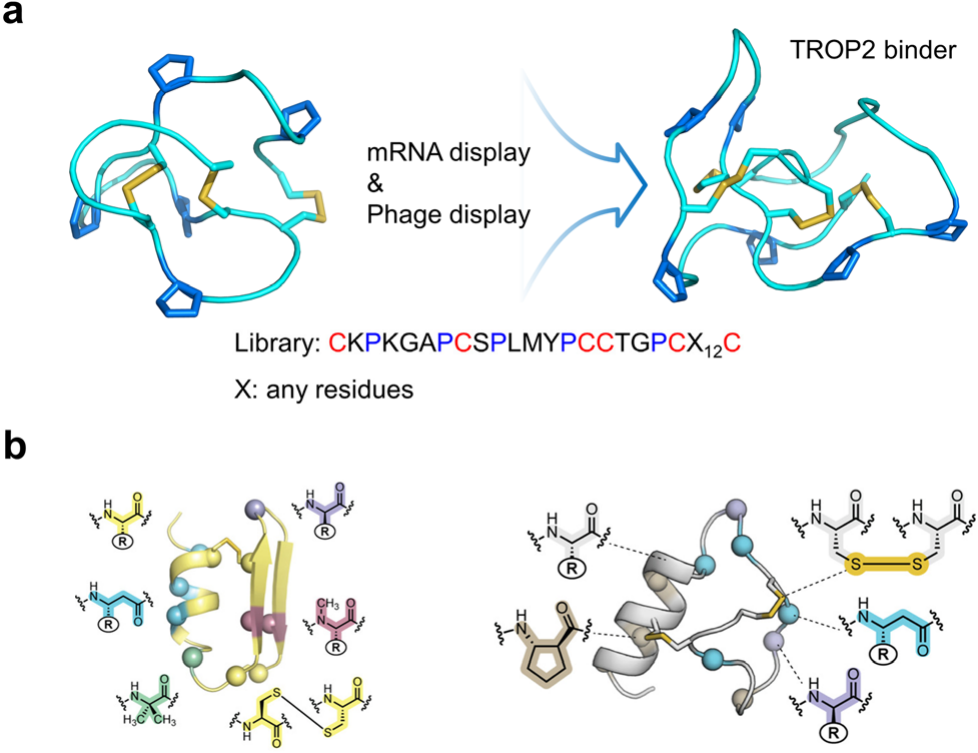
(a) Proline-mediated enhancement in evolvability of DRPs for discovering protein binders. (b) Heterogeneous-backbone proteomimetic analogues of a computationally designed DRP (left) and a natural antimicrobial peptide (right).

Among the emerging strategies developed to actively direct the oxidative folding of DRPs, proline-mediated scaffold engineering has proven particularly effective for enhancing foldability and evolvability (Fig. 7a).^119^ While DRPs such as cystine-knot peptides offer exceptional structural stability, their highly constrained topologies often limit sequence flexibility and reduce compatibility with high-diversity screening platforms. By strategically inserting proline residues into loop regions of model scaffolds like ω-MVIIA, the engineered variants achieved dramatically improved oxidative folding yields while preserving native disulfide connectivity and tertiary structure. More importantly, the scaffolds tolerated extensive sequence variation, enabling the construction of high-diversity libraries suitable for mRNA and phage display. Screening of these libraries yielded high-affinity binders against therapeutically relevant targets such as TROP2 and 4-1BB. In one case, a TROP2-binding DRP was incorporated into a chimeric antigen receptor (CAR) format and showed performance comparable to traditional antibody-derived CARs, with potential advantages in safety. These results highlight proline engineering as a practical strategy not only for promoting reliable oxidative folding but also for expanding the design space for robust and evolvable DRP scaffolds.

Another promising direction involves the design of heterogeneous-backbone proteomimetic analogues, in which portions of the peptide backbone are systematically modified using noncanonical residues (Fig. 7b).^120–122^ In one such study, an antimicrobial DRP was re-engineered to incorporate β^3^-amino acids and cyclic β-residues, introducing structural diversity while maintaining native disulfide connectivity.^120^ Although some analogues lost activity or failed to fold correctly, others retained both structure and function, while showing significantly enhanced protease resistance and reduced cytotoxicity. While the primary aim of this strategy was to improve pharmacological properties, these findings also demonstrated that careful backbone modification can also influence oxidative folding outcomes, pointing to its potential utility in future fold-directed design efforts.

A third strategy leverages machine learning (ML) to improve foldability in peptide library design.^123^ Rather than predicting detailed 3D structures, this approach focuses on identifying sequences with a high likelihood of folding into stable conformations. By training an ML model on data from yeast surface display and alanine-scanning experiments across a set of DRPs, researchers developed a predictive tool capable of estimating global foldability and identifying residues critical for proper folding. Guided by these predictions, they designed a new peptide library based on *de novo* designed DRP scaffolds. This library showed a higher fraction of well-folded members and yielded functional binders. Compared to conventional library designs, the ML-optimized approach improved folding outcomes without sacrificing sequence diversity. While still an emerging tool, foldability-focused machine learning offers a powerful and scalable complement to other strategies in peptide engineering and discovery.

These three approaches represent powerful and complementary strategies for actively directing the oxidative folding of DRPs. While they differ in their underlying mechanisms and primary objectives, each contributes to reducing the inherent stochasticity of disulfide bond formation. Proline engineering and machine learning approaches are particularly effective in enabling the design and screening of well-folded, diverse peptide libraries, supporting high-throughput discovery of functional molecules. In contrast, heterogeneous-backbone modification demonstrates how precise chemical editing can influence folding outcomes and improve pharmacological properties, though not explicitly applied to library design. These advances broaden the toolbox for oxidative folding control of DRPs, and lay the groundwork for future innovations in design, engineering, and discovery of next-generation DRP tools and therapeutics.

### Combination of fold-directing strategies

**Fig. 8:**
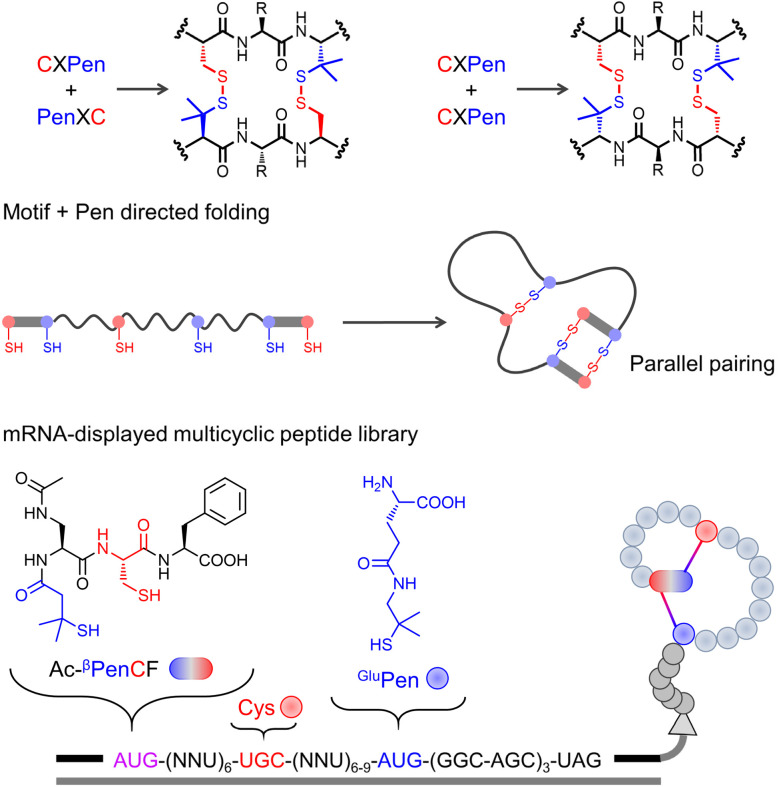
Dimeric pairing and folding of peptides with CXPen or PenXC motifs, and ribosomal incorporation of noncanonical disulfide-directing motifs for the construction of multicyclic peptide libraries.

While numerous strategies have been developed to direct the oxidative folding of DRPs, they are typically applied individually. However, the rational combination of orthogonal folding mechanisms holds great potential to overcome longstanding challenges in disulfide bond control and structural diversification. In our earlier work, building upon foundational CXC motif studies, we demonstrated a synergistic approach by integrating two mechanistically distinct strategies—CXC motifs and Cys–Pen orthogonal disulfide pairing—to direct the folding of complex multicyclic peptides (Fig. 8).^98^ This combination enables the spontaneous formation of predefined bicyclic and tricyclic topologies under mild redox conditions, yielding scaffolds that are structurally robust and tolerant to extensive sequence variation.^25,100,124,125^ CXC motifs, which promote dimeric ring formation, and Cys–Pen disulfide pairing, which operates with kinetic orthogonality, synergistically reduce disulfide isomer formation during folding.^6,98^ This allows predictable generation of specific conformations from fully reduced peptides without requiring sequence-dependent pre-folding. The resulting scaffolds, defined by the spatial arrangement and chemical properties of thiol-bearing residues rather than by primary amino acid sequences, represent a significant departure from the classical sequence-driven folding paradigm. By predefining disulfide connectivity through manipulating the arrangement of motifs and thiol-bearing residues, we constructed hyper-constrained peptide frameworks that fold with high structural precision and excellent yields. These designer scaffolds serve not only as stable templates for grafting bioactive sequences but also support ribosomal expression to design libraries.^103,106,109^ For example, we constructed an mRNA-displayed peptide library bearing noncanonical bisthiol motifs and Pen analogues, and successfully identified nanomolar affinity protein binders through screening (Fig. 8).^109^ This study also marks a critical advance toward directed evolution-compatible DRP libraries with folding outcomes dictated by chemically encoded design logic rather than by sequence-based folding rules.

In the future, the modularity and compatibility of the Cys–Pen system may enable its integration with other fold-directing strategies. For example, combining Cys–Pen orthogonality with CPPC or CPXXC motifs could generate more complex, fold-specific multicyclic architectures. Such combinations could expand the diversity of multicyclic topologies while further reducing folding heterogeneity through both spatial and directional constraints. Moreover, incorporating selenocysteine (Sec) into motif-directed peptide systems presents a particularly promising direction for achieving more precise and tunable control over folding pathways. Altogether, the future of DRP design may depend on the deliberate orchestration of multiple orthogonal mechanisms to create scaffolds that fold predictably, tolerate sequence diversity, and support functional evolution. As the field progresses, such combinatorial approaches will be key to unlocking the full potential of DRPs in chemical biology, diagnostics, and therapeutics.

## Opportunity and challenges

DRPs represent a highly valuable class of biomolecules with significant potential in both research and therapeutics. Among them, naturally occurring DRPs have been the most extensively studied, with many exhibiting potent bioactivities—most notably their ability to precisely target and modulate cell surface receptors such as ion channels, making them powerful molecular tools.^1,21^ In addition, natural DRPs possess inherent features such as exceptional resistance to proteolytic degradation and superior target specificity, making them outstanding candidates for drug development. Importantly, the emergence of actively directed oxidative folding strategies has greatly expanded the engineering potential of both natural DRPs and designed peptide scaffolds. By enabling high folding efficiency and fidelity, these approaches allow for unprecedented sequence flexibility. This tolerance for diverse sequence variation is critical for comprehensive drug optimization, which requires the simultaneous fine-tuning of multiple parameters, including target affinity and specificity, functional activity (inhibition or agonism), stability, circulatory half-life, and metabolic profile. Directed oxidative folding provides a robust foundation for this multifaceted optimization, enhancing drug-like properties and accelerating the clinical translation of engineered DRPs.

Natural DRPs also serve as versatile templates for generating novel ligands or binding molecules. Strategies such as epitope grafting or loop sequence randomization can yield libraries that target new protein interfaces.^126–128^ The increased sequence tolerance enabled by directed folding is especially crucial when incorporating substantial sequence modifications, as it improves the likelihood of maintaining the correct disulfide-bridged conformation in new variants. Notably, motif-directed oxidative folding represents a transformative advancement beyond conventional strategies.^2^ While natural scaffolds often permit only limited sequence variation in certain regions, motif-guided approaches enable the design of robust multicyclic peptide frameworks that can accommodate fully randomized sequences within defined structural architectures. This remarkable tolerance for radical sequence variation without compromising correct disulfide connectivity facilitates the construction of ultra-diverse peptide libraries. These libraries are particularly well-suited for the *de novo* discovery of ligands against challenging targets previously inaccessible to natural DRP-based approaches. Moreover, the ability to broadly modulate sequences while preserving correct folding is invaluable for systematically optimizing overall drug-like properties. The clinical potential of motif-directed DRPs is already becoming evident, with several candidates currently in clinical trials (ClinicalTrials.gov ID.: NCT06715020; NCT06713681),^129^ particularly for use as targeting components in radionuclide and fluorescent imaging probes for cancer diagnosis and surgical guidance.

Despite substantial progress, a central challenge in DRP engineering lies in navigating their vast sequence–structure space. Occupying a unique niche between traditional monocyclic peptides and small protein scaffolds such as affibodies or nanobodies, DRPs exhibit distinct structural characterics. Unlike scaffolds that rely on hydrophobic cores and sequence-dependent folding, DRPs derive their structural integrity primarily from covalent disulfide networks. This key distinction frees a greater portion of amino acid residues to serve functional rather than structural roles. As a result, despite being shorter in length than many protein scaffolds, DRPs can exhibit comparable—or even greater—functional sequence diversity. This is because their folding constraints are decoupled from specific folding-required sequence patterns, allowing more residues to directly participate in target binding. The potential diversity is staggering: a 30-residue DRP encompasses a sequence space of 20^30^, far exceeding the practical reach of any combinatorial library. Exploring this vast space to identify optimal binders is inherently difficult. A critical bottleneck lies in the dependence of the final optimized sequence on the initial template or library design. This inherent bias makes it difficult to determine whether the starting point was optimal, raising the question of whether the resulting peptide truly reflects the global optimum in terms of efficacy and developability. Addressing this limitation requires innovative library design strategies that leverage the structural plasticity of DRPs. By diversifying backbone topologies and cysteine frameworks—particularly through motif-directed folding—it is possible to generate libraries with greater structural heterogeneity that better match the complex surface features of protein targets. Maximizing combinatorial encoding within these defined frameworks significantly increases the likelihood of discovering DRPs with optimal structural complementarity and superior therapeutic properties.

Compared with small DRPs, many proteins exhibit significant higher disulfide bond density and greater structural complexity. For instance, members of the tumor necrosis factor receptor (TNFR) family feature multiple cysteine-rich domains, each stabilized by an elaborate network of intramolecular disulfide bonds.^130^ This structural intricacy both motivates and complicates the regulation of oxidative folding in larger proteins. Despite the considerable challenges, strategies developed to direct oxidative folding present significant opportunities for application to larger and more complex disulfide-rich proteins. Adapting these approaches could enable more advanced protein engineering, thereby broadening their functional utility. Advances in protein semisynthesis and total chemical synthesis now facilitate the site-selective incorporation of disulfide surrogates and constrained motifs into polypeptides that are difficult to modify genetically. These developments enhance the feasibility of implementing chemical strategies for protein engineering. Looking forward, the application of disulfide-directed folding strategies to larger proteins may lead to several potential outcomes, such as improved folding efficiency and stability, an expanded designable structural space for disulfide-rich proteins, and the possible development of engineered or stabilized biologics as novel therapeutics. If realized, these advances could help establish directed oxidative folding as a foundational methodology in protein engineering, potentially enabling more sophisticated manipulation of disulfide-rich proteins for both basic science and translational applications.

The therapeutic landscape is rapidly shifting from single-target agents toward multi-specific modalities, heralding a new era of innovation with bispecific (and multispecific) T cell engagers and chimaeras-based therapeutics.^131^ Engineered DRPs may hold significant promise in this area through two complementary strategies: (i) achieving multispecificity within a single scaffold by leveraging their inherent multi-loop architecture to engage multiple targets either simultaneously or individually, albeit with considerable design complexity, and (ii) modular assembly, in which distinct target-specific DRP domains are linked together to create multivalent constructs. Both approaches critically depend on directed oxidative folding. Only by ensuring the independent and accurate folding of each domain can misfolding and functional interference be avoided in such complex architectures. Furthermore, DRPs can enhance design versatility by being fused to antibodies or other protein domains to create advanced biologics,^132^ such as T cell engagers and proteolysis-targeting chimaeras. Robust directed folding is crucial in these cases, especially in expression systems where quality control mechanisms may not recognize misfolded DRP segments embedded in large fusion proteins, potentially leading to nonfunctional or aggregation-prone therapeutics. In addition, although fusion may compromise some advantages of free peptides (*e.g.*, deep tissue penetration), the retained compactness and flexibility of DRP domains allow unique tuning of binding kinetics, multivalency, and cooperativity, offering clear benefits over bulkier antibody-based constructs.

Cell therapies such as CAR-T are reshaping the treatments for cancer and autoimmune diseases. DRPs are emerging as promising candidates for compact, stable antigen-binding domains in chimeric antigen receptors (CARs).^119^ Our study indicated that DRP-based CAR-T cells can match the potency of conventional scFv-based CARs while significantly reducing cytokine release, thereby mitigating the risk of cytokine release syndrome (CRS), a major safety concern. Achieving this outcome critically depends on directed folding: the extracellular domain of CARs contains multiple structured elements, such as disulfide-linked hinges, and the DRP domain must fold accurately and independently without disrupting these components. This stringent requirement is uniquely fulfilled by motif-directed oxidative folding.

Compared with antibodies and their fragments commonly used in multispecific therapeutics and cell therapies, DRPs offer distinct structural and functional advantages. Their smaller size, rigidity, and unique paratope geometries enable them to recognize complex three-dimensional surfaces rather than linear epitopes, potentially affording deeper binding pockets, high binding specificity, unique binding kinetics, and differentiated therapeutic profiles that enhance efficacy or reduce side effects. Notably, motif-directed oxidative folding offers a key advantage over disulfide surrogate-based engineering strategies: it is inherently compatible with biological expression systems, operating without requiring post-synthetic modifications or specialized translational machinery. This makes it ideal for the production of complex therapeutics using cellular expression systems. While challenges remain in optimizing library design and multispecific formats, directed oxidative folding has resolved critical bottlenecks in production and functional performance. The integration of DRP engineering with next-generation therapeutic modalities, including T cell engagers, cell therapy, and targeted protein degradation, unlocks exciting new possibilities in medicine. This convergence would position DRPs as powerful next-generation molecular tools, expectedly driving a growing pipeline of candidates toward clinical translation and meaningful therapeutic impact.

## Author contributions

X. C. and C. W. collaboratively wrote the manuscript.

## Conflicts of interest

There are no conflicts to declare.

## Data Availability

No primary research results, software or code have been included and no new data were generated or analysed as part of this review.
